# “Warm,” “cool,” and the colors

**DOI:** 10.1167/jov.24.7.5

**Published:** 2024-07-08

**Authors:** Jan J. Koenderink, Andrea J. van Doorn, Doris I. Braun

**Affiliations:** 1 Justus Liebig University Giessen, Department of Psychology, Giessen, Germany; 2 KU Leuven, Experimental Psychology, Leuven, Belgium; 3 Utrecht University, Experimental Psychology, Utrecht, The Netherlands

**Keywords:** color appearance, color in art, phenomenology, warm–cool color quality

## Abstract

Participants judged affective cooler/warmer gradients around a 12-step color circle. Each pair of adjacent colors was presented twice (left–right reversed), all in random order. Participants readily performed the task, but their settings do not correlate very well. Individual responses were compared with a small number of canonical templates. For a little less than one-half of the participants responses or judgements correlate with such a template. We find a warm pole (in the orange environment) and a cool pole (in the teal environment) connected with two tracks that tend to have one or more gaps or weak, even inverted links. We conclude that the common artistic cool–warm polarity is only weakly reflected in responses of our observers. If it does, the observers apparently use categorical warm and cool poles and may be uncertain in relating adjacent hue steps along the 12-step color circle.

## Introduction

In the arts, there is a lot of talk about “warm” and “cool” colors, or chromatic relations (Dossie, 1758; Dunn, 1995; Koenderink, Braun, & van Doorn, 2021; Lewis, 2021; Quiller, 1989; Riley, 1995). What might that mean? Obviously, the concept of temperature,^1^ as familiar to physics, does not relate to colors, which are affective visual qualities. Even if we say of a flower that it is “red,” we refer to a color as a quality of an intended object (the thing we call “flower”) rather than some odd arrangement of elementary particles (Block, 1996; Byrne & Hilbert, 2003). For object colors, one has to conceive “warm” and “cool” not in thermodynamic or molecular dynamics terms, but as pointers to affects, or “raw feelings.”

In the past, two of the authors studied absolute judgements of the warm–cool quale (Albertazzi, Koenderink, & van Doorn, 2015). Here, we consider the sensitivity to warm–cool gradients. The reason is that artists often suggest that “warm” or “cool” colors as such do not exist, but that we have a sensitivity for gradients “cooler” or “warmer” in the vicinity of any given color. This sometimes has to do with spatial feelings. “Cooling” a patch of color is said to “push it back, whereas “warming” the patch would “bring it forward.” Indeed, such effects can be traced in experimental phenomenology (Koenderink, van Doorn, Albertazzi, & Wagemans, 2015). Thus, it may well be of interest to study warm–cool gradients next to “warm” or “cool” as (absolute) categories.

As said, the cool–warm polarity is an important one in the visual arts. The earliest reference we could find (Dossie, 1758) indeed refers to the arts, although the author was a chemist. In the sciences, the warm–cool polarity comes up in the study of color naming over cultures and languages (Lindsey & Brown, 2021). Recently there have been reports on a neuronal basis (Xiao, Kavanau, Bertin, & Kaplan, 2011). There are few empirical data in the literature from color science, perhaps because such studies are necessarily of a phenomenological and thus “subjective” nature. Relevant studies are Albertazzi et al. (2015), Knoblauch, Werner, & Webster (2023), and Specker et al. (2020).

What does it mean for a color to be felt as “warm” or “cool?” Or maybe it does not work that way and all we can say is that we perceive the blue of cornflowers as cooler than the red of poppies, or maybe for others the other way around. Are we judging colors or flowers? Is the intention color science or an expression of affective or aesthetic feelings (Arnheim, 1974; Goethe, 1810; Kandinsky, 1911; Klee, 2023)?

We feel artists probably know what they are doing, because they talk about warm–cool a lot when describing color compositions. But then, artists do not (generally) talk science. Art (æsthetics as Alexander Gottlieb Baumgarten (1750)’s science of “discourse of sensible representations”^2^) and science (as Aristotle (2018)’s “natural philosophy”) may be sisters (“Muses”) in the academic sense, but they hardly communicate.

Because the warm–cool dichotomy apparently is about colors—as different from flowers—we can make our understanding of colors count. For one thing, we know that hues come in a periodic linear sequence (Koenderink, van Doorn, & Gegenfurtner, 2019) and thus can be arranged in a circle. Hues are what count if we talk about warm or cold colors—for then we actually refer to colors as *hues*. So here are the alternatives as we see them:

• “Cool” and “warm” might be understood as a linear order relation “cooler” or “warmer” than. In that case, there are only two possibilities, namely clockwise (CW) or counterclockwise (CCW) about the color circle.
• “Cool” and “warm” might be understood in an absolute, categorical sense. Then there are colors that are warm or cool whereas their neighbors are the opposite, or indefinite. Because the color circle is closed one expects alternating “cold ranges” and “hot ranges.” Indeed, people might refer to oranges as “warm” as opposed to teals, which feel “cool,” whereas greens and purples are indefinite.
• Maybe there are colors that have no affective temperature at all (i.e., certain varieties of green, or purple), or maybe there are color pairs that do not stand in any “cooler” or “warmer” relation to each other. Then the present exercise might turn out to be abortive.

How to find out? The only ways are to look ourselves or to ask others. There is no physical apparatus or modus operandi that might help out. This is a human, affective thing, not to be addressed solely as problems in physics or chemistry (Goethe, 1810).

The scientific way is to ask a large number of observers and do statistics, so no single individual is to blame, the next best thing to objectivity. In such research, one takes it for granted that all (trichromatic) people are equal with respect to their perceptions. In this sense statistics is a way to truth. Such prior convictions are not based on facts. The phenomenological way is to look for ourselves so it is subjective by design. Only your own feelings are *meaning*, what you observe in others is *data*. Data and meaning are ontologically distinct. Ideally, data should be treated by fully objective means, detached from prior (subjective) notions. This results in the next best thing to meaning.

A productive way to proceed is to explore both approaches in parallel. This implies empirical phenomenology, keeping an open mind, in the sense of Husserl’s *Einklammerung* (“bracketing,” “epoché”) (Lübke, 1999). One “brackets” all knowledge and simply mindfully notes one’s awareness (Cogan, 2023). The world, the body (including the brain) and the mind cannot be pried apart without losing essentials. This knot is impossible to unravel.

How does one go about “asking people?” Obviously not by starting out to define what *you* “mean” (or that *they* “should mean”) by cool or warm. For that would introduce a *circulus vitiosus*. People should indicate what their meaning of warm or cool is by their observable actions. How to induce them to act in *your* intended way? There is no direct answer to that. Instead, one describes the operational methods and observations (data) in an objective manner. In the final instance the concept of cool–warm can only be operationally defined. In principle there are as many concepts as operationalizations.

## Experiment

### Setup

The setting was chosen to be as close to a generic user profile as possible. So we present the stimuli on an Apple notebook LCD screen, using the generic Apple LCD profile. It involves a gamma curve of the sRGB–type (exponent 2.4). The CIE xy chromaticity coordinates of red, green and blue are:

**Table:** 

Red	x = 0.6588,	y = 0.3338
Green	x = 0.3204	y = 0.6137
Blue	x = 0.1505	y = 0.0527

The chromaticity of the white point was *x* = 0.3126, *y* = 0.3291. The luminance of the (white) screen was 214 cd/m^2^.

Viewing was informal, binocular at convenient reading distance in a dark room. It seems unlikely that minor variations of this (in the range of “typical user interfaces”) might make much of a difference in this setting.

**Figure 1.**
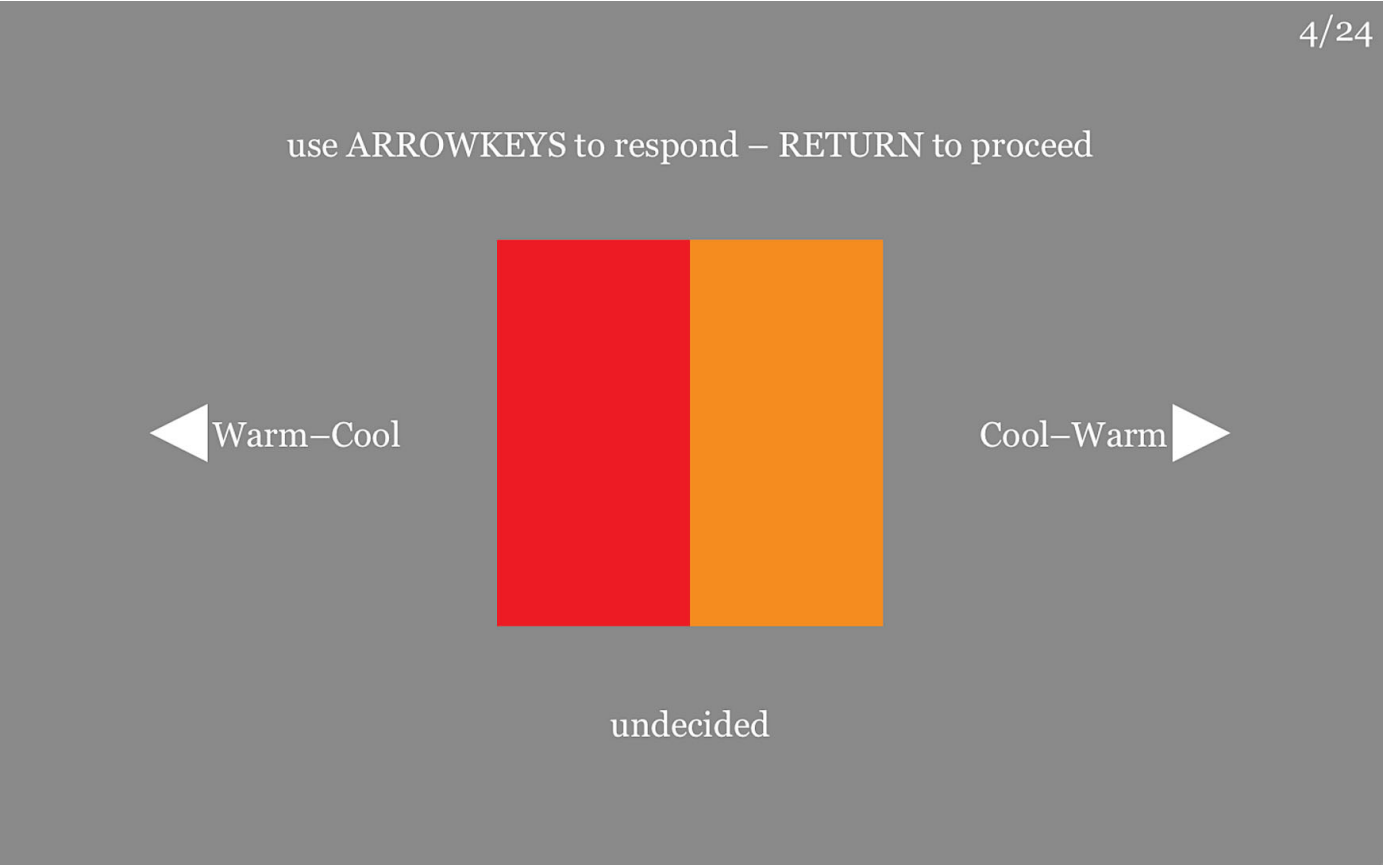
A screen dump from the experiment. This is trial 4 of 24 (top-right corner). The term ”undecided” appears at the start of the trial. The participant used the left-right arrowkeys to change “undecided” into “Cool–Warm” or “Warm–Cool.” “Undecided” does not count as a response. (Note that “Cool–Warm” implies “left cooler than right” and “Warm–Cool” implies “left warmer than right.”) After responding the return key triggers the next trial. The whole experiment does not take more than 5 minutes.

Participants were confronted with pairs of colored patches on the monitor screen (Figure 1). They had to use the keyboard arrows to indicate whether the left or the right patch appeared cooler or warmer. Of course, any pair was tried twice, with left–right reversed to bypass any left–right bias. Color pairs were adjacent steps from the usual 12-point color circle^3^:

• Red–orange;
• Orange–yellow;
• Yellow–leaf green;
• Leaf green–green;
• Green–sea green;
• Sea green–cyan;
• Cyan–teal (ice blue);
• Teal (ice blue)–blue;
• Blue–bluish purple;
• Bluish purple–magenta;
• Magenta–reddish purple;
• Reddish purple–red,

where we have borrowed a few convenient color terms from Wilhelm Ostwald (Ostwald, 1919a; Ostwald, 1919b; Ostwald, 1921).

### Participants

We tested 30 observers, about one-half were students of the university of Giessen, who were compensated for participation either with credit points or money. The other participants were lab members with different expertise with respect to color perception. Students were naïve to the purpose of the experiment.

Ages ranged from 19 to 75 (quartiles [21, 26, 33]); 77% were female and 23% male. All viewed stimuli binocularly, perhaps wearing personal correction. The screen was viewed in an informal setting (although the room was dark) from convenient reading distance.

Before starting the experiment color vision of each participant was tested with Ishihara’s 24-plates edition for color deficiency (Ishihara, 2018). We encountered several dichromatic observers, but these are not included in this study.

### Method

How do you explain the experiment without revealing what you mean by warm or cold yourself? That is evidently impossible. All you can do is point through examples of common bipolar categories and hope for the best.

We show (reproduced in the Appendix) pictures of pink sweets and green lemons, then suggest that mere colored patches of pink and green might already elicit feelings of sweet and sour. Many people buy that. We suggest this might be done as well with many other polar qualities, say heavy and light. Then we say “in this experiment it is about cool and warm,” but we do not show or explain anything. If you are persuasive in a nice way a few people might object, but in our case nobody did, or asked for further clarification. It is how we implement a (what we feel is) purely ostensive definition.

Then we explain the interface, as shown in the layout of Figure 1. Participants are confronted with pairs of colored patches and are invited to indicate (using the keyboard) whether the one or the other is cooler, or warmer. This works surprisingly well in the sense that participants are actually ready to *do it*.

We use a 12-step color circle and present all neighboring colors twice, once in left–right, once in right–left configuration. (This serves to rule out any left–right bias.) Thus a session involves 24 trials. Participants rarely take more than a few minutes. The fewer the better, because they need to respond on the basis of their visual intuition (their awareness) instead of reflective thought or introspection. (We explain to people that there is no such a thing as “the right answer”—the participant is always right. This is simply how we define “right”—it is what people *do*.)

### Results

**Figure 2.**
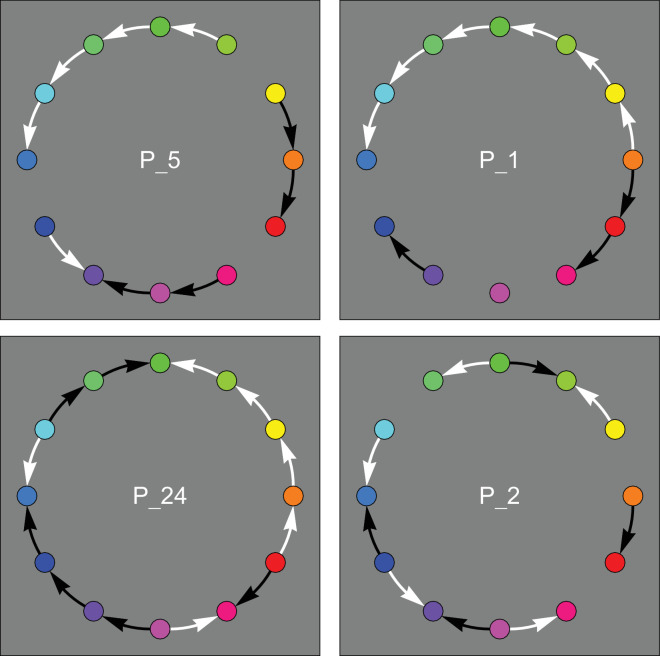
These are some typical individual results. (All results are gathered in Table 1 of the Appendix.) The white and black arrows run from warm to cool. If there is no arrow, the participant responded oppositely in the left-right reversed presentations of the same color pair. What to make of this? The examples at top and at bottom–left seem to show a common pattern, except for phase. Hot spots range from yellow to red; cool spots are predominantly teal. There seem to be reasonably well-defined tracks between the warm and cool spots. One track moves over green, the other over purple. Especially the track over green tends to be a clear sequence from the hot spot to the cold spot. The track over purple tends to show more gaps and apparent reversals. The example at bottom–right seems to be merely noise.

**Figure 3.**
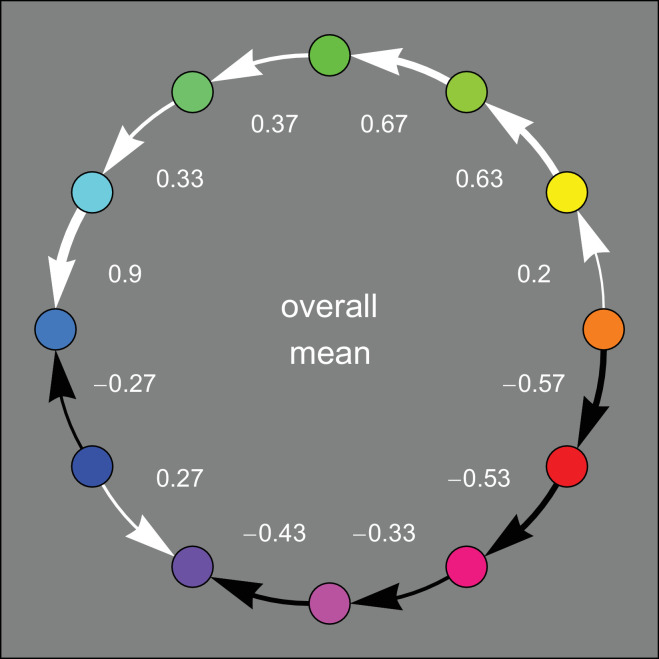
The overall average result. For clarity we use different colors to differentiate the CW and CCW directions. We also indicate the strength of the arrow both numerically and graphically through thickness. Thus the arrow that connects Yellow to Orange is so thin it is hardly different from a gap. In this average response the group considers yellow and orange as an undifferentiated fuzzy center.

Typical results are shown in Figure 2. In Figure 2, the arrows point in the direction “target is cooler than this location.” For the quantitative analysis we use the convention that CCW is positive (+) and CW negative (−). Thus the top-left case of Figure 2 reads (starting from orange) −0++++0+−−0−, where the “0” stands for “undecided.” In numerical analysis, we set ±1 for ± (this allows averaging (Figure 3), Fourier analysis (Figure 10) and so forth). This is how the “undecided” cases arise, they derive from a { +, −} response pair. (So “undecided” is not a response category, only + and − responses are allowed.) In the graphics we indicate + with a white CCW arrow pointing from warmer to cooler, whereas we indicate −1 with a black CW arrow, again pointing from warmer to cooler. This may take getting used to, but we find it is a convenient convention.

**Figure 4.**
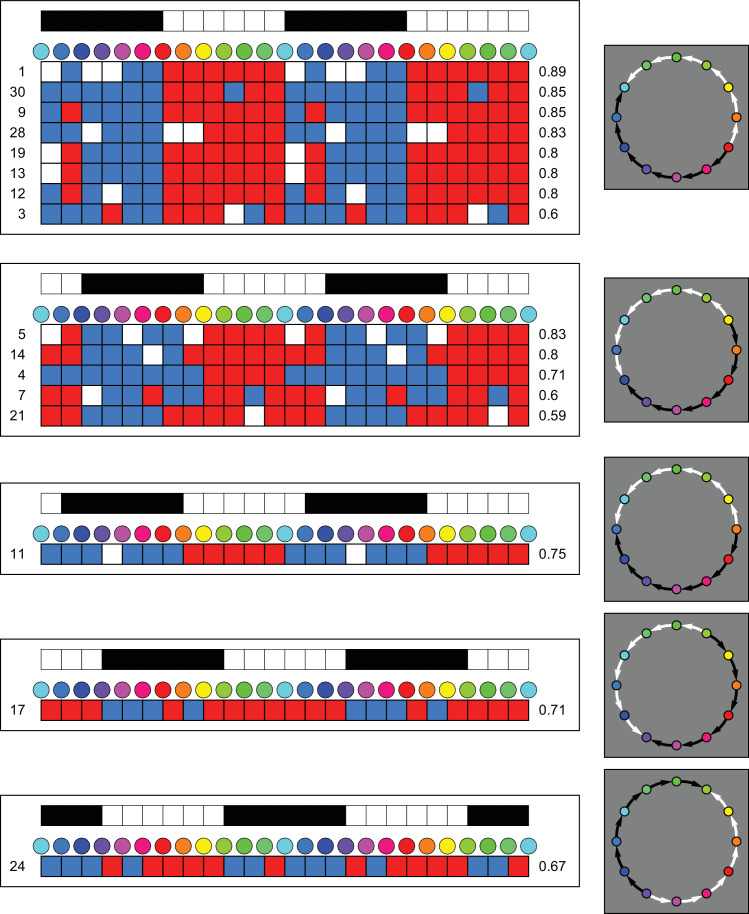
Detailed overview of settings of participants that correlated with the first order template. At the very top and in the right-side column we show the templates. The numbers at left are participant indices, the numbers at right are Kendall τ correlations with the templates. Colors indicate: blue, −1; white, 0; and red, +1.

**Figure 5.**
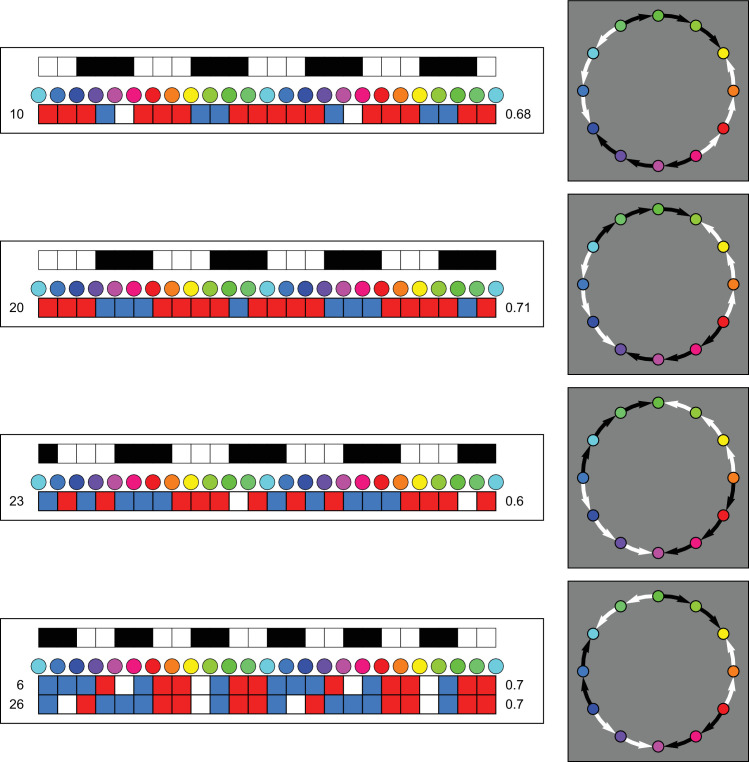
Detailed overview of settings of participants that correlated with the second- and third-order templates. The format is the same as in Figure 4. Note that the correlations tend to be lower than for the coarsest template.

**Figure 6.**
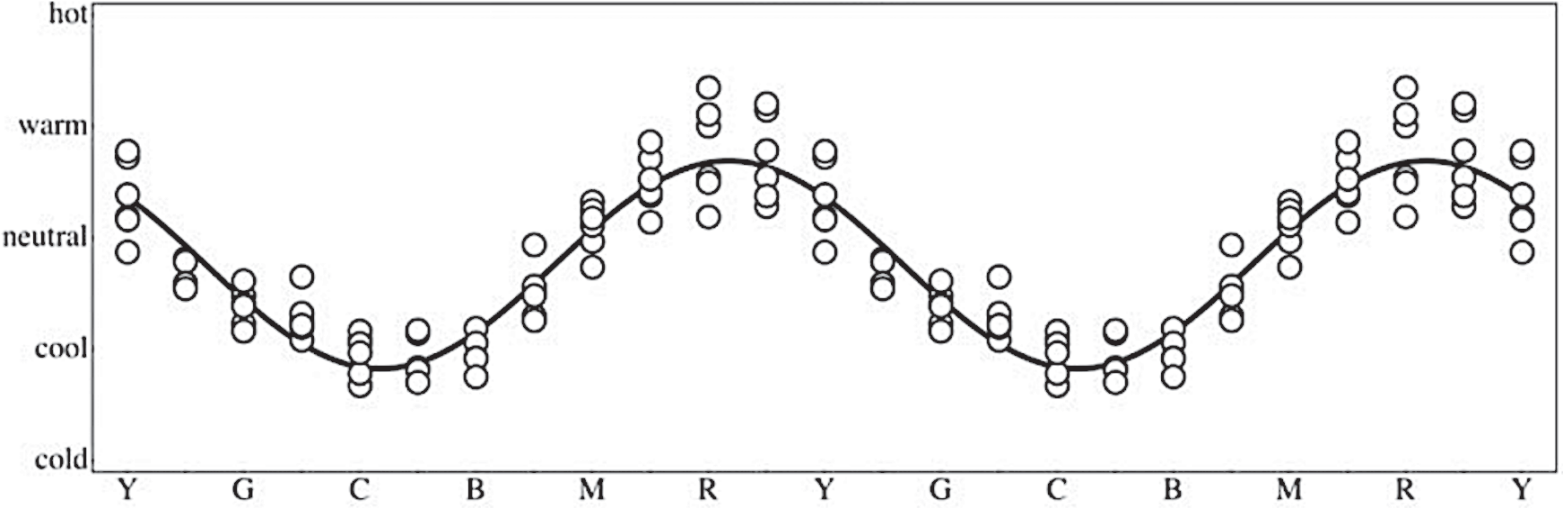
Results of previous measurements (Figure adapted from Albertazzi et al., 2015). Participants (37) ranked colors on a Likert scale (cold–cool–neutral–warm–hot). The fit is a sinusoid on the hue-scale of the color circle. The central maximum is at orangish red.

In Figure 2 top–left, note that there tends to be a long uninterrupted track from orange over green (thus CCW) to teal. There often is another long track (this one over purple, thus CW) from orange to teal, but it tends to be lacunary. Apparently the range from reddish purple to bluish purple is almost affectively isothermal and so is the stretch from blue to teal. There tends to be a fairly clear hot spot, whereas the corresponding cold spot is often more fuzzy. The example at bottom right seems to be merely noise. Indeed, various participants seem to have no well-defined differential warm–cool structure at all.

The gaps correspond with cases where the participant gave opposite responses to the same hue-pair in left–right and right–left presentations. (The frequency of such ties is close to 10%.) Of course, it might well be that some arrows are really gaps, or some gaps are really arrows, because when uncertain the participant simply does something at random. The way to deal with that would be to let the participant do numerous repeats. We refrain from that because many participants have wonderful memories, so repeats should be weeks apart to be mutually independent.

Our way to deal with this is to look at ensemble averages. This—in itself—is not a bad thing, but the advantage is also a disadvantage since you do not have individual data anymore. Yet it can hardly be doubted that people will often be mutually different in tasks like this. Anyway, we do not see a simple solution to this.

A view of the raw settings reveals vague patterns in a sea of—what we believe to be—random responses. Only repeated trials might establish this, but—as we remarked earlier—there cannot be such a thing as a truly independent repeat. You cannot step in the same stream (of awareness) twice. This pattern can be made more precise by a Fourier analysis, as presented in the Appendix, However, such a formal analysis glosses over the interesting local comparisons which are the focus of this study. Here we consider the raw data, which are gappy and shows occasional reversions.

It can be seen that many observers exhibit rather long tracks in both directions, and that most of them also show at least one gap somewhere. It may be instructive to just analyze a few instances picked at random to obtain an idea of what to expect.

One thing we note is that the two long tracks from orange to teal seem to underlie most of the action. However, on the way one meets with gaps and additional hot or cold centers. For instance, both green and purple may simply be passed en route, but they also occur as centers sometimes hot, sometimes cold. What we make of that is that the orange and teal centers are rather universal and strong (Koenderink & van Doorn, 2021), but that the passages from the one to the other are not major tracks. Halfway in between they weaken significantly, both near the green and near the purple.

The green and purple are not strong centers like orange or teal, they seem to just accidentally become centers now and then and may end up as hot as easily as cold.

### Analysis

In Figure 3 we show the overall result as a straight average. It suggests that there are two major centers, the set (yellow, orange) and teal. This also suggests that the track over purple is weaker—in the sense of more commonly broken—than the track over green.

For a detailed analysis we use rank correlations, opting for Kendall’s τ (Abdi, 2007) as a proper tool. With sequences of trinary values (CW, CCW, or tie) of length 12 (the number of cardinal points about the color circle) the statistical resolution is not very high.

It is not obvious how to check whether a response reflects pertinent data or whether it should be ignored as essentially random. Correlating responses (Kendal-tau rank correlation between the rows of Table 1 of the Appendix) pairwise reveals that only a fraction of 20% is significant at the 5% level. This cuts off some obvious ways one might start an analysis, like comparing individuals with the ensemble median.

A likely way to proceed is to compare the individual responses with a *template*. This is not an irrational way to go since the number of likely templates is really limited. (A formal Fourier analysis is presented in the Appendix.) The major tracks from orange to teal seem to be a kind of common feature. Thus, an obvious choice is the two-track pattern one spies at a close perusal of the raw responses. This has two runs of length six of CW and CCW responses. We may leave the phase open, so we correlate all with responses of the template in all phases.

The template might be regarded as the first-order term in a harmonic analysis based on random telegraph waves. It may be considered to be a kind of discrete Fourier analysis. We also consider higher order templates.

On the assumption that all responses are sequences of twelve random ±1 values that are mutually uncorrelated, a Kendall tau rank correlation of 0.67 or more has a 1/30 probability to occur. (An occasional zero value makes no difference to the second decimal place.) This implies that for our group of 30 participants one may expect on the average to have one of the responses exceed 0.67 due to mere chance. Thus, we consider only cases with correlations exceeding 0.67 as worthy of attention. (The conventional 5% level is 0.58.)

This reveals the following clusters of participants (Figure 4):

• One-third of the participants fails to correlate (at the 5% level) with any template. Mutual pairwise correlations range from −0.71 to +1. Only a fifth of these (just barely) reach the 5% level.
• Eight participants correlate with the first-order template that has the hot-spot at red (Figure 4 top). Correlations with the template range from 0.60 to 0.89.
• Five participants correlate with the first-order template that has the hot-spot at yellow (Figure 4 second from top). Correlations range from 0.59 to 0.83.
• Other cases involve a single participant. Three of these involve the first-order template with a hot spot at orange (third from top, correlation 0.75), leaf green (second from bottom, correlation 0.71) or bluish purple (bottom, correlation 0.67).
• There are three cases of correlation with a second-order template (all with different phases) and one case of a correlation with a third order one. See Figure 5. The rank correlations are much lower than the highest correlations overall (Kendall-tau over 0.8), so one should perhaps not attach too much attention to these cases. This also applies to some of the cases illustrated in Figure 4.

## Conclusions

**Figure 12.**
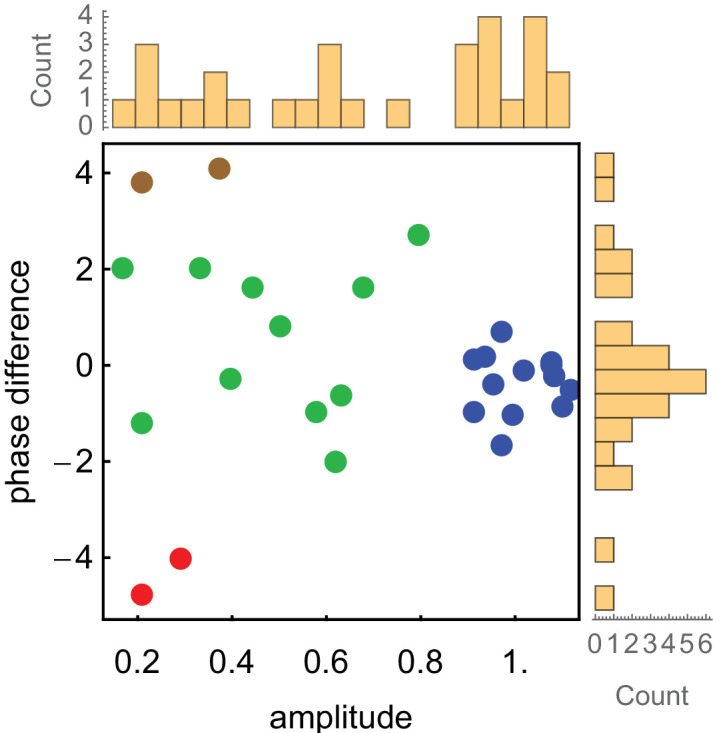
Histograms of amplitudes and phases for the dominant Fourier component of the individual participants and a cluster analysis. The largest cluster (blue) contains 14 items (some very close, thus hardly distinguishable).

Approximately one-half of the participants reveal a clearcut effect of “affective warm-cool gradient”. This group shows a qualitatively similar pattern of responses. A formal cluster analysis on the dominant Fourier component leads to the same result (Figure 12 in the Appendix). The major quantitative variations are shown in the upper two sub–groups detailed in Figure 4. (Perhaps ignoring the cases with relatively low correlations.)

The larger group of participants with an affinity to the warm-cool polarity recognizes a hot spot in the red region and a cold spot near the cyan region. Judging from the group data, they experience a gradual change from warm to cool by either track along the color circle. The location of the hot spot (and thus the cold spot) varies over participants though and the variation is perhaps surprisingly large. It runs from red to yellow.

The bottom line is that many people indeed distinguish “cooler than” or “warmer than” gradients over the color circle, although the locations of the hot and cold centers may vary a lot among persons. However, one should take note of the fact that about half of the group seems to be insensitive to such gradients.

In previous work (Albertazzi et al., 2015), we tested categorical judgements of affective temperature of colors. This yielded very systematic results (Figure 6). Essentially all participants readily performed the task. The hot spot was orangish red. We now see that it may be harder to judge affective warm–cool gradients. Only a little less than one-half of the current group of participants managed to do the task in the way that might be expected from the previous results. From the overall average (Figure 3) we glean that the hot spot is orange, or perhaps yellowish orange. This is indeed expected from the documented importance of the orange-and-teal palette often used in the visual arts and cinema (Koenderink, & van Doorn, 2021). Near the hot spot the sensitivity to gradients is low—which is expected if the participants act like they estimated a derivative of an absolute representation. However, the strengths of the tracks between the hot and the cold spots speak against this. That would implicate the greatest gradient sensitivity in the greens and purples, which is not at all obvious. Especially the track over purple seems to be weak and lacunary.

Of course, one needs to keep in mind that we have kept some parameters fixed in this study. For instance, there is a fixed background and the geometry of the stimuli was not varied either. A priori, any change in such parameters might make a difference, although we believe such to be minor. This expectation is partly based on the previous study (Albertazzi et al., 2015), where the colors were also varied in white and black content.

These findings throw some light on differences one finds in the literature on artistic and affective color. For instance, an author like Rudolf Arnheim (1974) seems to regard warm–cold mostly as a categorial perceptual judgement, whereas Johannes Itten (1974) seems to favor a more relative, gradient-based view. However, such authors often waver between these two extremes. Of course, we cannot know what our participants were actually doing. Maybe they never thought in terms of warmer and cooler as gradients, but only in terms of more or less orangish versus more or less tealish as mutually independent intensities. Then they would have warm and cool categories without having warm–cold gradients. And so forth. There are so many possibilities!

We conclude that although most observers are sensitive to the cool–warm polarity in an absolute sense, less than one-half are able to make coherent relative judgements of warmer than over a 12-step color circle.

## Figures and Tables

**Figure 7.**
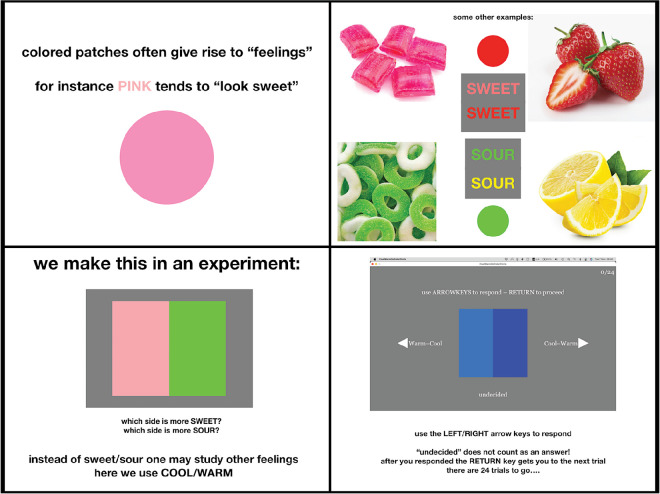
Four instruction sheets (read from left to right, top to bottom).

**Figure 8.**
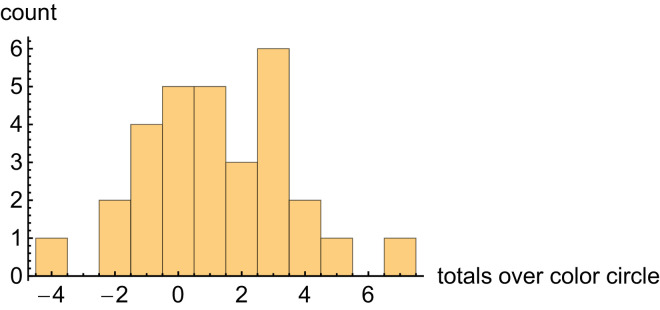
Histogram of the totals for the rows of Table 1.

**Figure 9.**
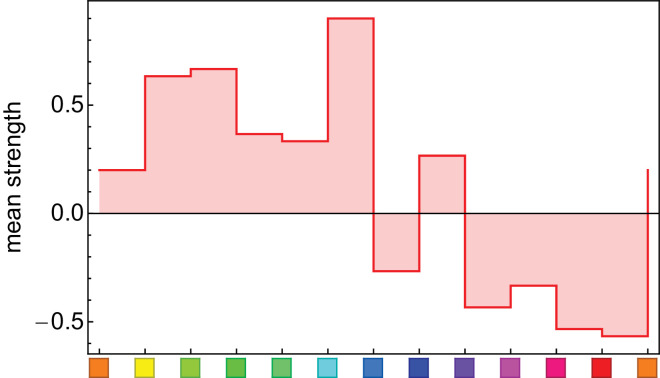
Means over the columns in Table 1.

**Figure 10.**
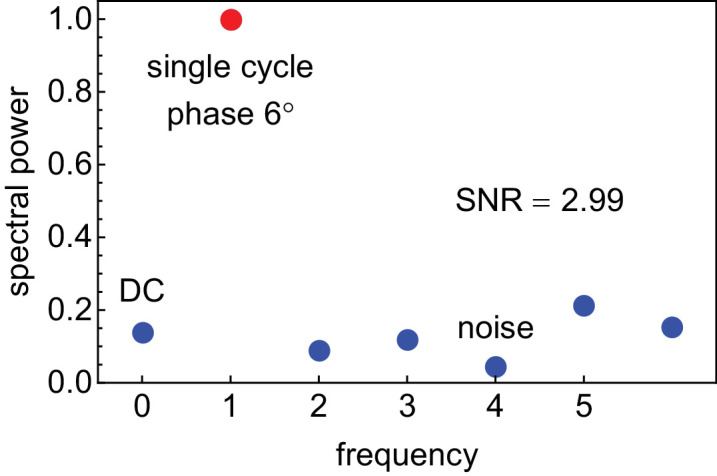
The Fourier power spectrum of the mean response (that is Figure 9). The red dot indicates the fundamental (single cycle) component. There is no indication of harmonics of any order. There is simply a white noise background.

**Figure 11.**
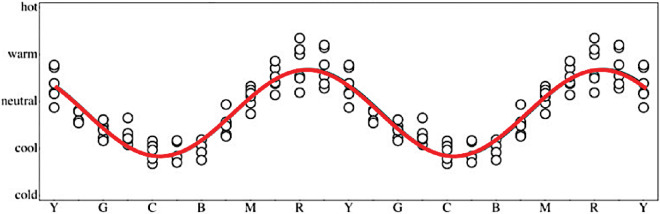
The integrated dominant component superimposed (offset and amplitude adjusted appropriately, plotted in red) on the categorical data. The phase yields an almost perfect fit (the original black fit (Figure 6) is covered by the red Fourier fit).

**Figure 13.**
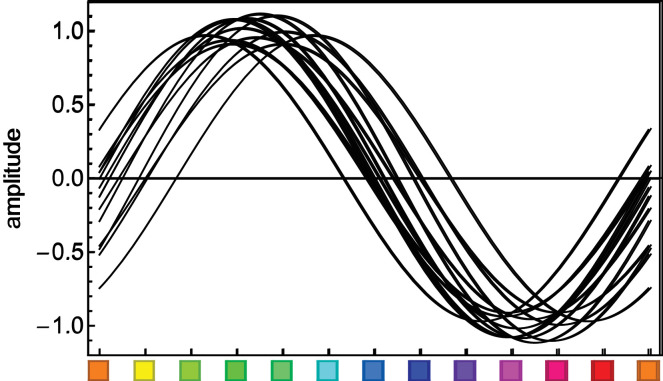
The fundamental Fourier component for the individual participants in the largest cluster (blue points in Figure 12). This representation masks the fine structure of the raw data (which is why we put it in an Appendix), but it very well brings out overall trend. It shows that the present differential data are not at odds with the categorical data of Albertazzi et al. (2015) (Figure 6).

## Footnotes

1 Not to be confused with the notion of “color temperature!” The “hotter” a radiant object (temperature in the sense of thermodynamics), the cooler its color (“color temperature,” a colorimetric term).

2 Æsthetica ...est scientia cognitionis sensitivæ.

3 RGB coordinates: $\left(\left\{1,0,0\right\},\left\{1,\frac{1}{2},0\right\},\left\{1,1,0\right\},\left\{\frac{1}{2},1,0\right\},\left\{0,1,0\right\},\left\{0,1,\frac{1}{2}\right\},\left\{0,1,1\right\},\left\{0,\frac{1}{2},1\right\},\left\{0,0,1\right\},\left\{\frac{1}{2},0,1\right\},\left\{1,0,1\right\},\left\{1,0,\frac{1}{2}\right\}\right)$. Conventional hex-codes (#FF0000, #FF8000, #FFFF00, #80FF00, #00FF00, #00FF80, #00FFFF, #0080FF, #0000FF, #8000FF, #FF00FF, #FF0080).

## Appendix

### Instructions

Figure 7 shows the formal instruction sheets every participant was asked to read before starting the session.

### All data

In Table 1 we show what we consider the raw data. All that is lost is the left–right order for the single trials: the values represent the mean over left–right reversed trials. The notation has been explained at the start of section Results.

**Table 1.** The horizontal parameter is the hue step *h*, which refers to the pair {*h*, *h* + 1}, where *h* = 0 labels orange and *h* = 1 labels yellow. The participants have been (arbitrarily) sorted by the length of the longest run of +1 responses. Each item is the mean of two responses on mutually left–right reversed presentations. Positive stands for CCW, negative for CW, and zero for a tie.

	0	1	2	3	4	5	6	7	8	9	10	11
P_17	1	−1	1	1	1	1	1	1	1	−1	−1	−1
P_14	−1	1	1	1	1	1	1	1	−1	−1	−1	0
P_19	1	1	1	1	1	1	0	1	−1	−1	−1	−1
P_13	1	1	1	1	1	1	0	1	−1	−1	−1	−1
P_12	1	1	1	1	1	1	−1	1	−1	0	−1	−1
P_9	1	1	1	1	1	1	−1	1	−1	−1	−1	−1
P_1	1	1	1	1	1	1	0	−1	0	0	−1	−1
P_25	−1	1	1	1	1	1	−1	1	1	−1	−1	−1
P_18	1	1	1	−1	1	1	1	0	1	−1	1	1
P_11	−1	1	1	1	1	1	−1	−1	−1	0	−1	−1
P_10	1	1	−1	−1	1	1	1	1	1	−1	0	1
P_28	0	0	1	1	1	1	−1	−1	0	−1	−1	−1
P_24	1	1	1	−1	−1	1	−1	−1	−1	1	−1	1
P_21	1	1	1	1	0	1	1	1	−1	−1	−1	−1
P_20	1	1	1	1	−1	1	1	1	1	−1	−1	−1
P_5	−1	0	1	1	1	1	0	1	−1	−1	0	−1
P_4	−1	−1	1	1	1	1	−1	−1	−1	−1	−1	−1
P_30	1	1	1	−1	1	1	−1	−1	−1	−1	−1	−1
P_23	−1	1	1	1	0	1	−1	1	−1	1	−1	−1
P_22	1	1	1	0	−1	1	−1	1	−1	0	−1	−1
P_15	1	−1	0	−1	1	1	1	−1	1	−1	−1	−1
P_8	−1	1	1	1	−1	−1	1	1	−1	−1	1	1
P_7	−1	−1	1	1	−1	1	1	1	0	−1	−1	1
P_3	1	1	1	0	−1	1	−1	−1	−1	1	−1	−1
P_29	−1	1	0	−1	0	1	−1	1	−1	1	1	−1
P_27	−1	1	−1	0	−1	1	−1	−1	−1	1	1	0
P_26	1	1	0	−1	1	1	−1	0	1	−1	−1	−1
P_16	−1	0	1	1	−1	0	−1	1	−1	1	1	0
P_6	1	1	0	−1	1	1	−1	−1	−1	1	0	−1
P_2	0	1	−1	1	0	1	−1	1	−1	1	0	−1

The participants have been sorted with respect to the lengths of the longest run of +1 values (remember the hue sequence is periodic!). We grant that the table is not easy to parse. We tried several alternative sorting schemes before deciding on the present one, but saw no way for improvements.

Note that the total over the color circle (sum of all values in a row of the table) is rarely zero. Figure 8 shows a histogram.

In Figure 9 we show the means over the columns of the table. This perhaps best reveals the overall structure of the data. The standard deviation is about 0.8 and varies only little over hues. Although the magnitude of the standard deviation does not mask the overall pattern, it severly limits reasonable inferences relating to the precise shape of the stepwise curve.

### Comparison with categorical data

We showed categorical data in Figure 6. Although the present experiment was aimed at the nature of relative judgements over the color circle, it is natural to ask whether the categorical data can be obtained by integration of the differential ones. A numerical integration is an option, but the presence of an imbalance (as apparent from Figure 8) poses problems. A better way is to use a Fourier technique.

In Figure 10 we show the Fourier power spectrum of the mean data (Figure 9). Note the presence of a DC–component and noise at high frequencies. However, the lowest frequency of interest (one cycle over the color circle) is clearly dominant. It is the phase of this component that is of major interest. An integration implies a 90° phase shift. As illustrated in Figure 11 this yields an almost perfect fit.

The quality of this fit should not be overestimated, for it is partly due to chance. This is evident from a look at the individual responses. Many are too noisy to show the same fundamental component (Figure 12). Indeed, there may not be a fundamental component at all. Apparently, the influence of random variations cancels out in the mean (Figure 11).

The signal to noise ratios for the fundamental component vary from near zero (no signal detectable) to about thirteen (well defined fundamental component). In Figure 12 we show a scatterplot of amplitudes and phases. A cluster analysis yields what is immediately visually apparent. There exists a cluster (blue) of 14 cases that stand out through a narrow range of phases and high amplitudes. (These are the participants {1, 4, 5, 9, 11, 12, 13, 14, 17, 19, 21, 25, 28, 30}. The signal to noise ratios for these cases ranges from 3.8 to 12.7, with quartiles {4.8, 7.5, 8.9}.

Thus, only about half of the participants (14 of 30) shows the expected warm–cool reactions through their differential judgements. This is different from the categorical data, where virtually all participants yielded similar responses.

In Figure 13 we show the fundamental components of this group. Apparently, the phases range over about two steps of the 12-step color circle The median phase is −0.21, a reddish orange. The range is −1.67 a purplish red to +0.67 an orangish yellow.

There is a good agreement with the template matching analysis (Figure 4). Participant 25 is a member of the main cluster, but its correlation with the first-order template just barely reaches the 5% level, so it is not included in Figure 4. Participants 3, 7, and 24 do correlate with the first order template, but are not in the main cluster. In all cases (3, 7, 24, 25), the spectra are atypical with structureless, strong high frequency content. We can conclude little more than that these cases are due to random cause. The two methods (template matching and Fourier analysis) both implement a form of noise immunity, but do so in qualitatively different ways.

Of course, the analysis done for the fundamental component can be repeated for any frequency. By a signal-to-noise criterion (signal-to-noise ratio of >3) one indeed finds several contenders (Table 2).

**Table 2.** Number of significant (signal-to-noise ratio > 3) frequency components. There are no overlaps between the frequency groups.

Frequency	Number of cases
0	1
1	14
2	2
3	5
4	0
5	1
6	5

The fundamental component (frequency 1 with 14 cases) immediately jumps out.

The next remarkable group is frequency 3 with five cases. It might be thought that these are harmonics (in the sense of having a phase relation) of the fundamental. That is not the case though, because there is no phase relation. The phases of these components vary widely and can even be in counter phase. This indicates that the frequency groups—except for the fundamental frequency 1—do not represent homogeneous groups of participants.

This is also the case for the highest frequency. That is why these frequencies fail to appear in the Fourier powerspectrum of the mean response (Figure 10). We consider it appropriate to ignore anything but the fundamental. This is in accord with the template matches discussed in the main text. For the latter we could ascertain that any higher-order components might well be due to chance. The Jaccard ratio for the sets of participants found through the template and the Fourier method is 0.76 (Jaccard 5% significance limit is 0.5), thus there is excellent concordance.
